# Identification and Structural Analysis of Amino Acid Substitutions that Increase the Stability and Activity of *Aspergillus niger* Glucose Oxidase

**DOI:** 10.1371/journal.pone.0144289

**Published:** 2015-12-07

**Authors:** Julia Marín-Navarro, Nicole Roupain, David Talens-Perales, Julio Polaina

**Affiliations:** Instituto de Agroquímica y Tecnología de Alimentos, CSIC, Paterna, Valencia, Spain; CNR, ITALY

## Abstract

Glucose oxidase is one of the most conspicuous commercial enzymes due to its many different applications in diverse industries such as food, chemical, energy and textile. Among these applications, the most remarkable is the manufacture of glucose biosensors and in particular sensor strips used to measure glucose levels in serum. The generation of ameliorated versions of glucose oxidase is therefore a significant biotechnological objective. We have used a strategy that combined random and rational approaches to isolate uncharacterized mutations of *Aspergillus niger* glucose oxidase with improved properties. As a result, we have identified two changes that increase significantly the enzyme's thermal stability. One (T554M) generates a sulfur-pi interaction and the other (Q90R/Y509E) introduces a new salt bridge near the interphase of the dimeric protein structure. An additional double substitution (Q124R/L569E) has no significant effect on stability but causes a twofold increase of the enzyme's specific activity. Our results disclose structural motifs of the protein which are critical for its stability. The combination of mutations in the Q90R/Y509E/T554M triple mutant yielded a version of *A*. *niger* glucose oxidase with higher stability than those previously described.

## Introduction

Glucose oxidase (GOX) characteristically produced by species of *Aspergillus* and *Penicillium* catalyzes the oxidation of β-D-glucose using molecular oxygen as electron acceptor, to yield D-gluconolactone and hydrogen peroxide. The enzyme belongs to the glucose-methanol-choline (GMC) oxidoreductase family. It is also classified within Auxiliary Activities family 3 (AA3) in the carbohydrate active enzymes (CAZy) database [1]. GOX activity is dependent on the cofactor flavin-adenine dinucleotide (FAD), which is transiently reduced along the reaction mechanism. D-gluconolactone is spontaneously hydrolyzed to yield gluconic acid [2].

GOX has a wide range of industrial applications in diverse sectors such as food, pharmaceutical, energy and textile [3]. As a food additive, GOX has remarkable preservative, antioxidant, and stabilizer properties. Hydrogen peroxide generated by GOX action has anti-microbial effect. Subsequent hydrogen peroxide decomposition results in net oxygen consumption with antioxidant and stabilizing effects. In the textile industry, GOX is used for bio-bleaching and in oral care products as antimicrobial agent. However, the major commercial use of GOX is in biosensors for blood sugar testing.

Among glucose oxidases, *Aspergillus niger* GOX is the most commonly used enzyme for industrial applications. Early studies with this enzyme showed that it was a glycosylated dimeric protein, which had a mass of about 160 kDa and used FAD as cofactor [4, 5]. Its structure has been extensively analyzed [6–8]. The protein is a dimer of two identical units. Each monomer contains two domains, one consists of a five-stranded β-sheet sandwiched between a three-stranded β-sheet and three α-helices, and the other is composed by a large six-stranded antiparallel β-sheet supported by six α-helices. The FAD cofactor is not covalently but tightly bound to the first domain. The two units of the dimer are connected through hydrophobic and hydrophilic contacts, the latter including salt bridges and hydrogen bonds.

While being used for many applications, intrinsic properties of GOX, particularly thermal stability and optimum pH for activity, are limiting factors. Therefore, directed evolution approaches have been used to improve enzyme performance [9–11]. Alternatively, available detailed information about the GOX structure makes possible to carry out enzyme engineering through rational design. This has been used to increase catalytic efficiency of the enzyme which also resulted in a moderate increase of thermal stability [12]. In this work we have used two alternative approaches to select new GOX variants with improved stability. One consisted of random mutagenesis, followed by a screening for enzymes with higher residual activity after incubation at restrictive temperature. The other was based on rational design, aiming to introduce stabilizing salt bridges. The combination of both approaches resulted in two new GOX variants with improved thermal stability and a third one with higher specific activity. Moreover, this study provides information that reveals the importance of certain elements of the protein structure in the overall stability activity of the enzyme.

## Materials and Methods

### Expression in yeast of the *Aspergillus niger* gene coding for GOX

The GOX-encoding gene, which lacks introns [13], was cloned from genomic DNA of *Aspergillus niger*. The DNA was purified by a standard procedure (see supplemental material) from strain CECT 2775 obtained from *Colección Española de Cultivos Tipo* (www.cect.org). The GOX coding sequence, excluding its signal peptide, was amplified by PCR with oligonucleotides JM823 (TAC*GCTAGC*AGCAATGGCATTGAAGCCAGC) and JM824 (CAC*AAGCTT*TCACTGCATGGAAGCATAATCTTC), containing *Nhe*I and *Hind*III restriction sites, respectively (underlined). PCR was carried out with a proofreading polymerase (Phusion, Thermo Sci), according to the specifications of the manufacturer. Plasmid pSTRD-Bgl1 [14] was used to construct plasmid pSSP-GOX (S1 Fig) for the expression of GOX in *S*. *cerevisiae*. In pSSP-GOX, the GOX coding sequence is fused to the *STA1* signal peptide from *Saccharomyces cerevisiae var*. *diastaticus* and is under the control of the galactose-inducible *GAL10/CYC1* promoter (see supporting information for details). *Saccharomyces cerevisiae* BY4741 (*MAT*a *his*3 *leu*2 *met*15 *ura*3) was used as the host strain.

### Mutagenesis

Site directed mutagenesis was carried out by PCR as previously described [15] using plasmid pSSP-GOX as the template. Oligonucleotides used as primers are listed in Table 1. Double mutants were obtained in a second round of mutagenesis, using the templates and primers indicated in Table 2. In all cases, a proof-reading polymerase (Phusion, Thermo Sci) was employed. The PCR-amplified DNA with the target mutation once cloned was sequenced to ensure the absence of additional undesired mutations within the GOX coding sequence.

**Table 1:** 

Primer/sequence^a^	Resulting plasmid
**NR966**/*A*AACTGGCCCGCAACATCTCC **NR967**/AGTAGCGGCAGCCTGACCG	pQ469K
**NR964**/*GAT*AGCGCCTGGACTGAGTACATCC **NR965**/ATCGGCATCATACGCGAGGTTATC	pL500D
**NR916**/*AGG*GCTGAGCGTGCTCGCG **NR917**/GAGGGAGTAGGCGGCCACATTG	pQ142R
**NR914**/*GAA*AAAATTTCGGATGCTATCTTGGAAG **NR915**/CGCCATGGCATAGAACACCG	pL569E
**NR962**/*AG*AACCGCGCTGATCCGCTC **NR963**/ATTGTTGGTAGCGAGCTCCACG	pQ90R
**NR960** */GAA*CACTTCCGTCCTAACTACCATG **NR961**/CGGGATGTACTCAGTCCAGG	pY509E
**NR956**/*AA*GCCGGACCCCGTGACAC **NR957**/*T*GACAGTACCATTGGTACCATGACAG	pH172K
**NR958**/*G*ACGAAGACCAAGTTCGCTCCG **NR959**/CAAGGTGTTGGGGAACATGGAC	pH220D
**NR869**/*AAG*TTCGCCTACGACCCTCAG **NR870**/GTGAAGGTAGGGGTCCTTGTC	pH447K
**NR946**/*AAA*GGTCAGGCCGCTTGGTTC **NR947**/TCCGGCACCAGCAGAGG	pQ345K
**NR837**/*G*TCCGCTCCGGAAATGGTCTCG **NR838**/CAGCGCGGTTTGATTGTTGGTAG	pI94V
**NR835**/*T*CTGGACTCACCACCGCTGC **NR836**/CAGACCTCCACCAGCGATG	pT30S
**NR922**/A*T*GCAAATGTCGTCCCATGTCATG **NR923**/AGGAGGAATAGAACCATCAATGACACG	pT554M

^a^italic, underlined characters represent mutated nucleotides.

**Table 2:** 

Double/triple mutants	Template	Primers	Resulting plasmid
Q469K/L500D	pQ469K	NR964 and NR965	pQ469K/L500D
Q142R/L569E	pL569E	NR916 and NR917	pQ142R/L569E
Q90R/Y509E	pY509E	NR962 and NR963	pQ90R/Y509E
H172K/H220D	pH220D	NR956 and NR957	pH172K/H220D
T30S/I94V	pI94V	NR835 and NR836	pT30S/I94V
T554M/Q90R/Y509E	pQ90R/Y509E	NR922 and NR923	pT554M/Q90R/Y509E
T554M/Q142R/L569E	pQ142R/L569E	NR922 and NR923	pT554M/Q142R/L569E

Random mutagenesis was carried by PCR with a polymerase without proofreading activity (Paq 5000, Aglilent Technologies) with oligonucleotides JM823 and JM824. The PCR product was digested with *Nhe*I / *Hind*III and re-cloned into pSSP-GOX. *E*. *coli* was transformed with the resulting ligation product. The population of transformant cells was enriched by growing the transformed culture overnight in liquid LB medium supplemented with ampicillin (25 μg/ml). Restriction analysis of the pool of plasmid DNA molecules isolated from this culture (named pSSP-GOX*) indicated that at least 90% of the plasmids contained a GOX insert.

Plasmids encoding the wild-type GOX or different mutant versions of the enzyme, were used to transform *S*. *cerevisiae*. Transformant colonies were selected in SD (minimal) medium with all auxotrophic requirements except uracil.

### GOX activity and thermal stability assays

A plate assay was carried out to determine activity in yeast colonies grown on solid medium. Transformant colonies were grown on petri dishes of YPD (complete) medium supplemented with 0.5% galactose and 2.8 mM *O*-dianisidine. Colonies transformed with the same plasmid lacking the GOX encoding gene were used as a negative control. The plates were incubated at 80°C for 30 minutes, a condition that causes inactivation of the wild type enzyme, and then cooled back to room temperature. To assay for GOX activity, 10 ml of reaction mix P, containing 10 μg/mL horseradish peroxidase (HRPO, Sigma) and 90 mM glucose (Sigma) in 50 mM phosphate buffer pH 6, were added per plate. After 30 minutes of incubation at 37°C, the plates were washed with deionized water and kept at 4°C for 20 minutes. Active transformants were detected by the formation of an orange halo.

Activity in liquid medium (culture supernatant) was assayed as follows. Colonies were firstly grown on SD plates supplemented with requirements lacking both uracil and leucine, to force the maintenance of the plasmid in high number of copies as an effect of the defective *leu2*d gene present in the plasmid [16]. Individual colonies were inoculated in triplicate in 500 μl YPD supplemented with 0.5% galactose and cultured at 30°C with agitation. After 48 hours the cells were separated by centrifugation and the culture supernatant was used to assay GOX activity and thermal stability. All culture samples were split in three aliquots: one was kept on ice, to determine initial activity, and the other two were incubated at 60°C for two different times (t_1_ and t_2_) before chilling on ice. For each set of experiments, wild-type and mutant enzymes were assayed in parallel under the same conditions. Incubation times (t_1_ and t_2_) were selected to render a residual wild-type activity of ca. 40% and 25%. Subsequently, the samples were incubated at 37°C in a reaction mix L, containing 85 mM glucose, 12 μg/mL HRPO, 0.17 mM *O*-dianisidine in 50 mM pH 6. The reaction was stopped by addition of 0.2 M HCl and cooling on ice. Absorbance at 400 nm was measured and the amount of oxidized glucose was determined by interpolation in a standard curve. The glucose standard curve was prepared as follows: known amounts of glucose were incubated with 12 μg/mL HRPO, 0.16 mM *O*-dianisidine and 25 μg/ml of commercial GOX from *A*. *niger* (Sigma, G7141), for 30 minutes at 37°C. The reaction was stopped with 0.2 M HCl and the absorbance at 400 nm was measured. A_400_ was plotted versus glucose concentration. GOX activity was determined as the amount of oxidized glucose per unit of time.phosphate buffer

### SDS-PAGE analysis of GOX and determination of specific activity

Yeast transformants were grown for 2 days in 5 mL of liquid SD medium supplemented with auxotrophic requirements except uracil and leucine. These cultures were used to inoculate 100 ml flasks containing 50 ml of YPD with 0.5% galactose at a cell density equivalent to OD_600_ = 0.014. The flasks were incubated for 2 days at 30°C with orbital shaking at 250 rpm. After this time, the culture medium was separated from the cells by centrifugation and concentrated 30–60 fold by ultrafiltration through a membrane with a 20 KDa cutoff (Thermo Sci). An aliquot from the concentrated sample was incubated with 3 U/μl EndoH (New England Biolabs) in 50 mM phosphate buffer pH 6, 90 mM β-mercaptoethanol and protease inhibitor cocktail (Complete, EDTA-free, Roche) for 16 hours at 37°C. A control sample was treated in the same conditions but without EndoH. Both deglycosylated and control samples were analyzed by SDS-PAGE, as previously described [14]. The bands corresponding to GOX in EndoH-treated samples were quantified using an image analysis software (Quantity One, BioRad), and their intensities (M) were recorded. GOX activity (A) was determined in the concentrated, EndoH-untreated samples. Specific activity is defined as the ratio of the enzyme activity and its mass (A/M). These ratios were normalized taking the value corresponding to wild-type GOX as 1.

### Bioinformatic tools

Multiple sequence alignment was carried out with Clustal W [17]. Structural homology modeling was performed with I-TASSER [18]. Structural analysis and in silico site-directed mutagenesis was carried out with Pymol (Schrödinger). Sterically-permitted, energetically favored orientations of the possible rotamers of some mutations were explored with Swiss-pdb viewer (http://www.expasy.org/spdbv/).

## Results

### GOX sequence analysis

Oligonucleotides used as primers for PCR amplification of the GOX coding region were designed on the basis of the GOX sequence with code X16061 in GenBank. However, the sequence of the DNA fragment resulting from the amplification showed higher similarity to another GOX sequence, GenBank KJ774107.1. The amplified GOX differed from KJ774107.1 only in two nucleotides and encoded the same amino acid sequence. Therefore, in this communication, we consider *A*. *niger* wild type GOX gene sequence that corresponding to GenBank KJ774107.1. The encoded amino acid sequence (AID16306.1) shows two changes (V167T and K282E) with respect to the GOX sequence reported in crystallographic structures (PDB codes: 1GAL, 1CF3, 3QVR, 3QVP). These residues are located on the protein surface, are fully exposed to solvent and do not show significant interactions with other residues in the protein.

### GOX random mutagenesis and screening for mutants with improved thermal stability

**Fig 1:**
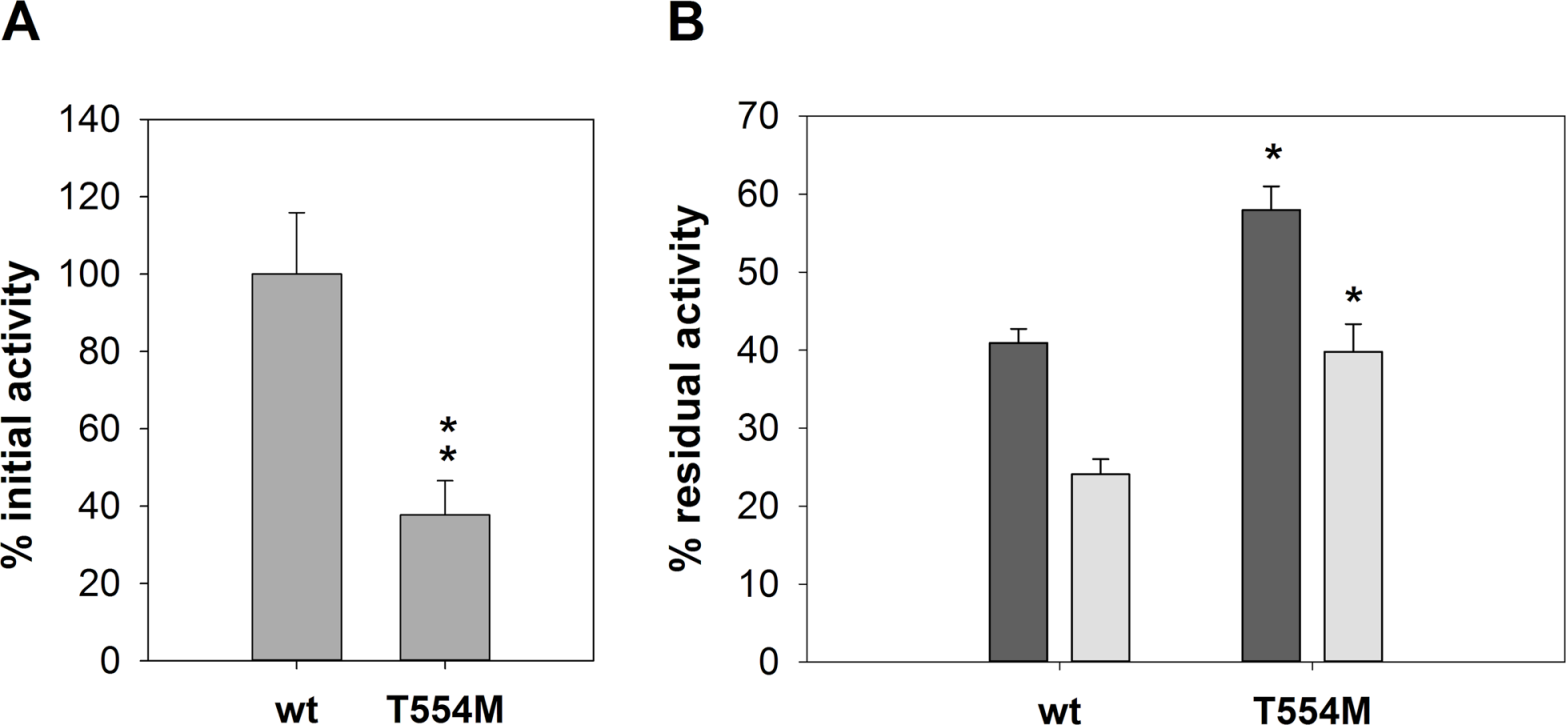
Thermal stability of T554M mutant obtained by random mutagenesis. (A) Relative initial GOX activity in the culture medium of the yeast transformants. (B) Residual GOX activity after incubation at 60°C for 45 (dark grey bars) or 80 (light grey bars) minutes. Error bars represent standard deviation of triplicates. Significant differences (p < 0.05 or p < 0.01) with the wild-type enzyme are indicated by one or two asterisks, respectively.

A library of 2800 *S*. *cerevisiae* clones expressing randomly mutated GOX coding sequences were subjected to the semi-quantitative plate assay, using as a control a *S*. *cerevisiae* transformant with the wild-type version of the gene. 274 clones showed GOX activity after incubation of at 80°C. Thermal stability of GOX produced by these clones was further analyzed. They were individually cultivated in liquid medium where the expression of the enzyme was induced by the addition of galactose. The crude enzyme preparation from each clone was heated at 60°C for 45 or 80 minutes and the residual activities (R1 and R2 respectively) were determined. Clones expressing wild type GOX gave R1 ca. 40% and R2 ca. 25%. (Fig 1). R1 and R2 from the different clones were compared to those of wt-GOX. Only one mutant showed a significant increase in thermo-resistance. The positive clone was subjected to three consecutive rounds of single colony isolation on plates of solid medium to ensure homoplasmicity. Plasmid DNA was then recovered from the transformant and sequenced, revealing a single mutation corresponding to the substitution T554M. This plasmid was used to retransform *S*. *cerevisiae* and the phenotype of the resulting transformant was tested again. While GOX activity produced by the T554M yeast transformant was around 40% compared to the wild-type, the mutant enzyme showed a higher resistance to thermal denaturation, with 40% and 60% higher R1 and R2, respectively, compared to the wild-type version (Fig 1).

### Rational design of mutations aimed to improve GOX thermal stability

**Fig 2:**
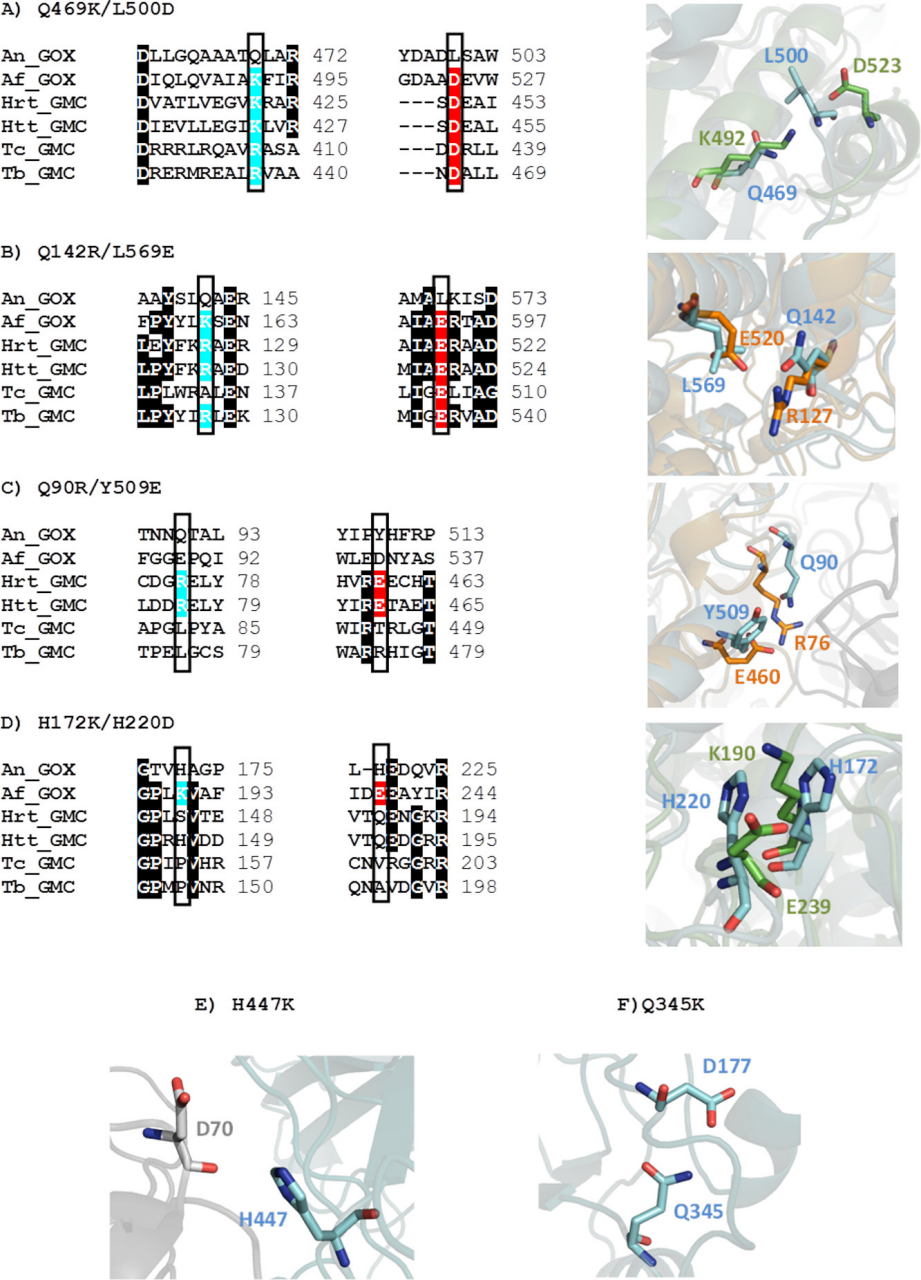
Mutations designed to introduce new salt bridges in *A*. *niger* GOX. (A) Q469K/L500D; (B) Q142R/L569E; (C) Q90R/Y509E; (D) H172K/H220D; (E) H447K; (F) Q345K. In A-D, sequence alignments with homologous enzymes from thermo-tolerant organisms are shown. Sequence codes are as follows: An_GOX: GOX from *A*. *niger* (Uniprot code P13006); Af_GOX: GOX from *A*. *fumigatus* (Uniprot code BOXU64); Hrt_GMC: glucose-methanol-choline oxidoreductase from *Halorubrum tebenquichense* (Genbank code WP_006628503.1); Htt_GMC: glucose-methanol-choline oxidoreductase from *Haloterrigena thermotolerans* (Genbank code WP_006648055.1); Tc_GMC: Glucose-methanol-choline oxidoreductase from *Thermomonospora curvata* (Uniprot code D1A2Y2); Tb_GMC: Glucose-methanol-choline oxidoreductase from *Thermobispora bispora* (Uniprot code D6Y5M6). Residues involved in the predicted salt bridges in An_GOX-homologous enzymes are highlighted in blue (cationic partner) and red (anionic partner). Panels on the right show details of An_GOX structure (PDB code 1CF3) and homology-based models of Af_GOX (green) and Htt_GOX (orange). An_GOX residues to be mutated and those involved in putative salt bridges in the homologues are shown. Panels E and F display the position of two single mutations. The residue to be mutated and the putative partner to form a salt bridge are shown. The two subunits of An_GOX structure are depicted in grey and blue.

The role of superficial salt bridges in protein thermal stability is well documented [19–21]. A set of site-directed mutations was designed to introduce new salt bridges that might increase the thermal stability of the enzyme. Some of the mutants (Q469K/L500D, Q142R/L569E, Q90R/Y509E and H172K/H220D) were designed based on sequence alignments and structural overlapping with GOX-related enzymes from thermophilic organisms (Fig 2A–2D). In regions of the protein where the sequence was poorly conserved among homologues, as in the dimer interface, we sought for pairs of residues in two loops distant in the primary sequence but close enough in the quaternary structure to allow the formation of a salt bridge when mutated to charged residues. Mutant H447K was designed to introduce two symmetrical, intermolecular salt bridges at the dimer interface, between K447 and D70 (Fig 2E). The mutation Q345K aimed to create a salt bridge with D177 (Fig 2F). Double mutant T30S/I94V, previously reported as thermoresistant [10] was also generated for the sake of comparison.

**Fig 3:**
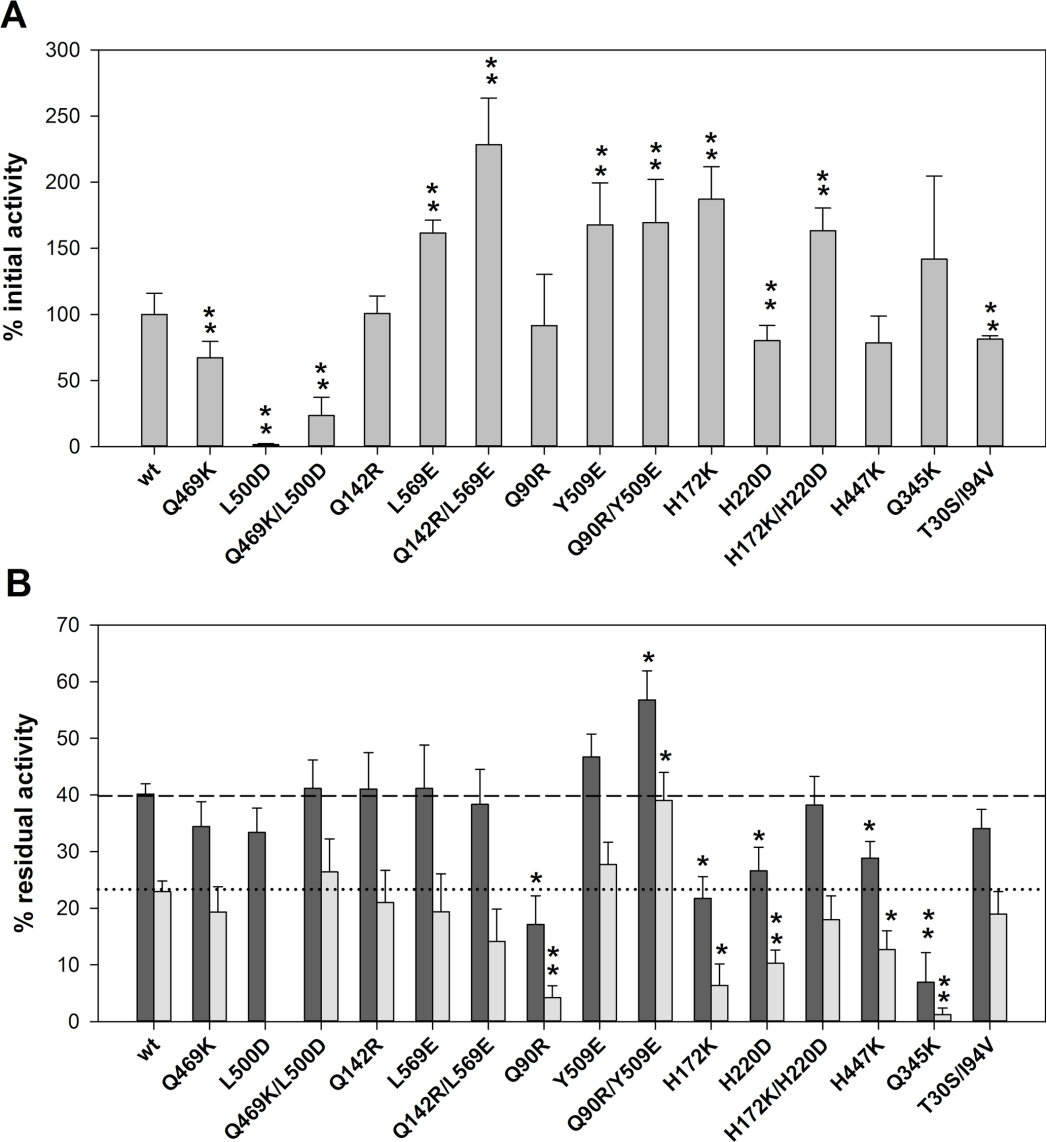
Thermal stability of the mutants obtained by rational design. (A) Relative initial GOX activity in the culture medium of the yeast transformants. (B) Residual GOX activity after incubation at 60°C for 45 (dark grey bars) or 80 (light grey bars) minutes. Error bars represent standard deviation of triplicates. Significant differences (p < 0.05 or p < 0.01) with the wild-type enzyme are indicated by one or two asterisks, respectively.

Thermal stability of the aforementioned mutants was studied by determining R1 and R2 (Fig 3A). Initial GOX activity (before thermal treatment) of the different mutant enzymes was also analyzed (Fig 3B). Q469K/L500D, Q142R/L569E and their corresponding single mutants did not show significant differences in thermal stability compared to the wt-GOX, while initial activity was lower for the Q469K/L500D series and higher for L569E and Q142R/L569E. On the other hand, H447K and Q345K showed a similar initial activity but higher thermal sensitivity, with R1 values 30% and 80% lower than the wild-type enzyme, respectively. The double mutant H172K/H220D did not show significant differences in thermal stability compared to the wild type enzyme although both of the corresponding single mutants (H172K and H220D) were more thermo-sensitive. In these experimental conditions, mutant T30S/I94V did not show a significant increase of thermal stability either. Finally, Q90R/Y509E mutant showed a 40% and 70% higher R1 and R2, respectively, compared to wild-type enzyme, whereas the single mutant Q90R had an increased sensitivity to thermal denaturation, with R1 and R2 values 60% and 80% lower than wt-GOX, respectively. The mutation Y509E did not cause a significant change in the thermal stability of the enzyme. Both Y509E and Q90R/Y509E transformants showed higher GOX activity than the wild-type GOX strain while the Q90R transformant GOX initial activity was not significantly different.

### Specific activity of GOX mutants

**Fig 4:**
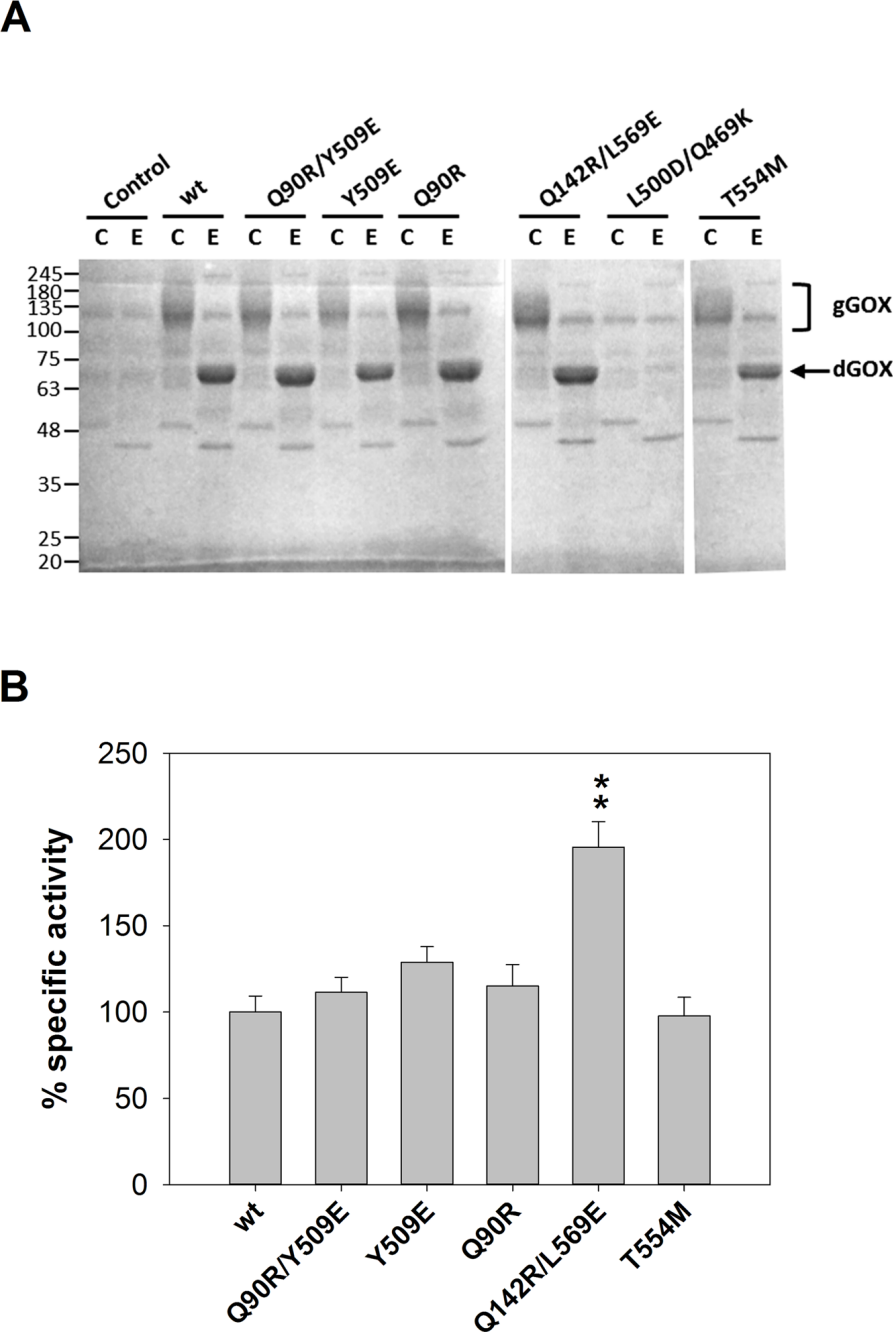
Analysis of glycosylation pattern and specific activity of selected GOX mutants. (A) Proteins released to the culture medium were analyzed without (C) or with (E) EndoH treatment. GOX was identified as a differential band compared to a yeast control transformed with the same plasmid lacking the GOX gene. Migration of the deglycosylated GOX (dGOX) in the E lanes is indicated by an arrow and that of the glycosylated GOX (gGOX) in the C lanes is shown by a bracket. (B) Relative intrinsic activity of GOX mutants. Error bars represent standard deviation of analytical triplicates. Significant differences (p < 0.01) with the wild-type enzyme are indicated by asterisks.

In principle, differences in GOX activity among the *S*. *cerevisiae* transformants (Figs 1B and 3B) could be due to either variation in the expression levels of the enzyme or changes in the intrinsic enzyme's catalytic efficiency. We addressed this question with the mutants that showed higher thermal stability, namely, T554M and Q90R/Y509E (Figs 2A and 3A). As a control we also analyzed two double mutants, Q142R/L569E and L500D/Q469K, which showed no difference in thermal stability (Fig 3A). GOX activity (A) was determined for the different transformants and the mass of enzyme (M) was measured by scanning deglycosylated protein bands in SDS-PAGE (Fig 4). In the gels, GOX protein was identified as a differential band in the electrophoretic pattern of the transformant strains compared to a control containing only the expression vector. The amount of enzyme secreted by the double mutant L500D/Q469K was under the limit of detection (Fig 4A), suggesting that the low activity value detected in the culture medium (Fig 3B) was mainly a consequence of a reduced protein concentration. This may reflect a problem in the folding and/or secretion of this mutant enzyme by *S*. *cerevisiae*. No significant changes were found in specific activity (A/M) of the rest of mutants except for Q142R/L569E which showed an almost 2-fold increase, compared to the wild-type enzyme (Fig 4B). The pattern of hyperglycosylated forms of all these mutants was similar to that of the wild-type enzyme (Fig 4A).

### Combined effect of selected mutations

**Fig 5:**
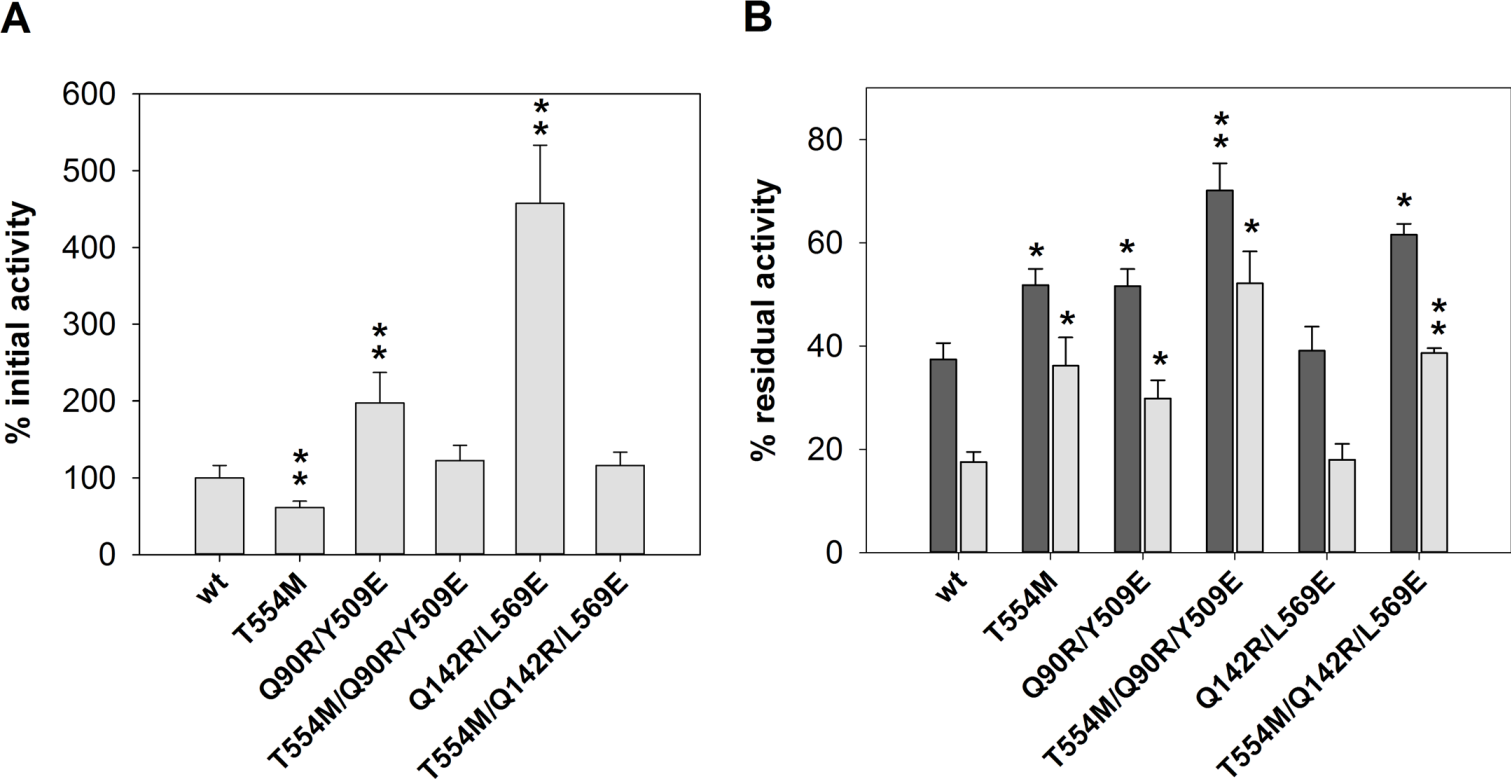
Thermal stability of the enzymes with combined mutations. (A) Relative initial GOX activity in the culture medium of the yeast transformants. (B) Residual GOX activity after incubation at 60°C for 25 (dark grey bars) or 45 (light grey bars) minutes. Error bars represent standard deviation of triplicates. Significant differences (p < 0.05 or p < 0.01) with the wild-type enzyme are indicated by one or two asterisks, respectively.

Mutations that improved either specific activity or thermal stability were combined aiming to generate new enzyme variants with better properties. Triple mutant T554M/Q90R/Y509E was designed to bring together the stabilizing effects of T554M (Fig 1) and Q90R/Y509E (Fig 3). Triple mutant T554M/Q142R/L569E intended to combine thermal stability of T554M and increased specific activity of Q142R/L569E (Fig 4). The reason was that despite of its thermal stability, T554M protein was produced in lower amount than the wild-type. The result of the analysis of the combined mutations is shown in Fig 5. Enzyme preparations were incubated at 60°C and residual activities were determined after 25 and 45 minutes of incubation (R1 and R2 in this experiment). Wild type GOX gave 37% and 18% R1 and R2 values, respectively. Triple mutant T554M/Q90R/Y509E performed significantly better than parental mutants T554M and Q90R/Y509E. Residual activities R1 and R2 increased ca. 1.9 and 3.0- fold compared to the wild-type enzyme. Triple mutant T554M/Q142R/L569E kept the thermal stability of the T554M mutant but the initial activity found in the culture medium was restored to the values found for the wild-type enzyme.

## Discussion

Glucose oxidase is one of the most relevant industrial enzymes because of its diverse applications, among which the most remarkable is its use in glucose biosensors. Therefore, strategies directed to improve GOX stability and activity have a significant biotechnological interest. The research described in this communication, in which we have used a combined approach involving random mutation and structure-based protein rational design, is framed in this context. In addition to improve the performance of the enzyme, our aim was to uncover structural features that could be targets for further improvement.

**Fig 6:**
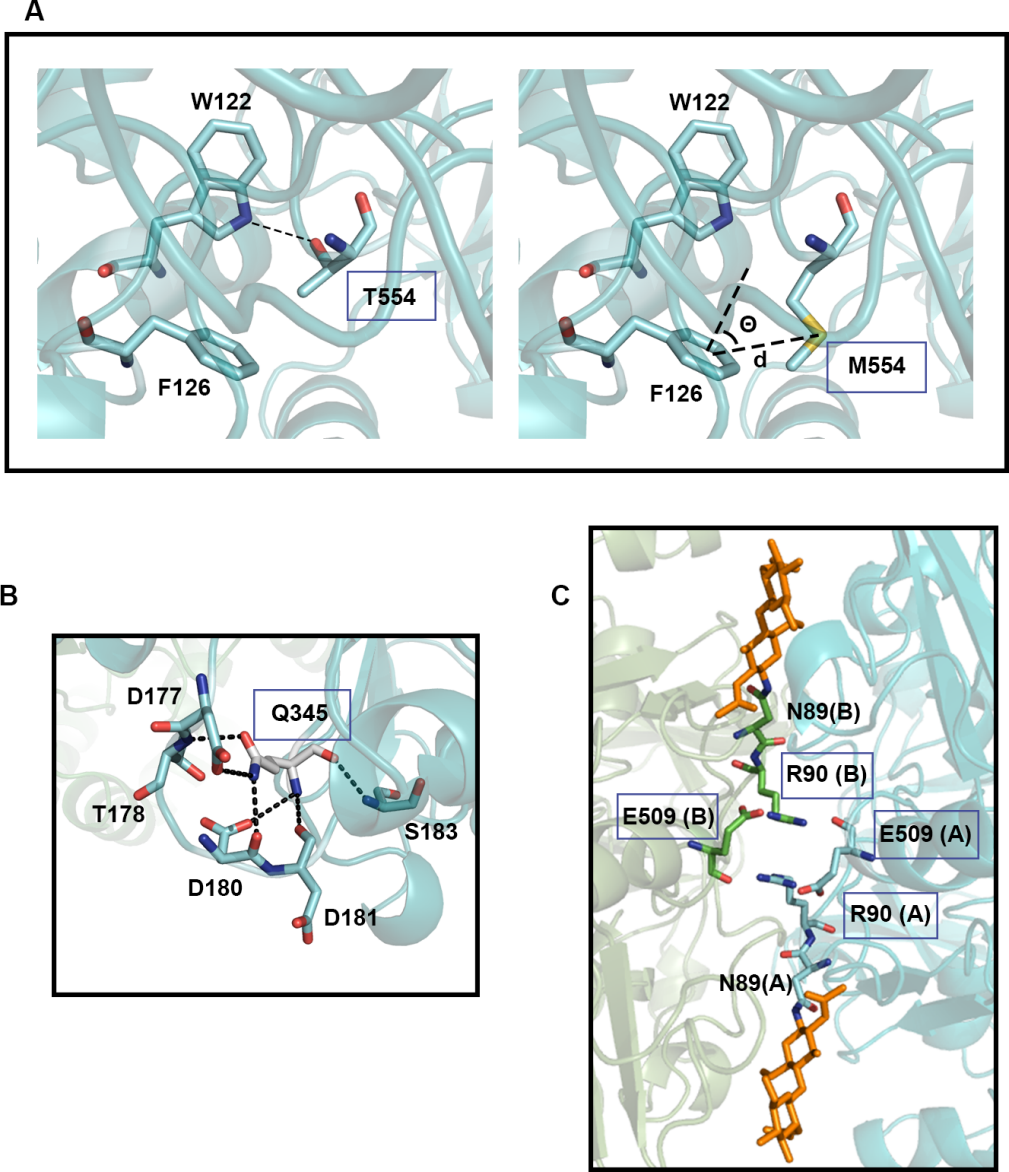
Structural detail in the vicinity of residues critical for GOX stability. Relevant interactions are depicted with dashed lines. (A) T554 in wild-type enzyme (left panel) and M554 in T554M mutant (right panel). Θ = 60°; d = 5 Å. (B) Q345 in wild-type enzyme. (C) R90 and E509 in the double mutant Q90R/Y509E. The subunit of origin is indicated in parenthesis. N-acetyl-glucosamine modification is colored in orange.

Selection of mutants after random mutagenesis, for which we stablished a rather high threshold (ie. presumptive mutant clones showing a weak effect were discarded) displayed an unexpected effect resulting from Thr to Met substitution at position 554. The analysis of the protein structure shows that mutation T554M disrupts a hydrogen bond between T554 and W122 (Fig 6A, left panel). Instead, M554 is likely involved in a sulfur-pi motif, involving the interaction of the sulfur atom with an aromatic group [22]. In Met-aromatic ring interactions, the intermolecular distance between the sulfur and the aromatic ring center is ~ 5.5 Å, with a preferential orientation of 30–60° between the sulfur atom and a normal vector to the plane defined by the aromatic ring at the ring center. In the modeled structure of the T554M mutant, the geometry of the side chains of M554 and F126 is compatible with the formation of a Met-aromatic motif (Fig 6A, right panel). The energy associated with the sulfur-pi interaction (4.2–12.6 kJ/mol) is comparable to the stabilization provided by a salt bridge (4.2–13.4 kJ/mol), and higher than that associated with a hydrogen bond (1.3–6.3 kJ/mol) [22]. The relevance of the Met-aromatic motif in protein stabilization is suggested by its relatively high prevalence, being present in ~ 33% of the resolved protein structures. Moreover, several mutations of Met residues interacting with aromatic residues are associated with pathological conditions, suggesting that the loss of the Met-aromatic motif results in a protein malfunction. Met substitution in other cases resulted in thermal instability and improper protein folding. Our results strongly suggest that the introduction of Met-aromatic motifs can be a strategy to achieve protein stabilization.

Some mutations designed to increase GOX stability showed an opposite effect. Mutations H447K and Q90R, located at the dimer interface (S2 Fig) caused a sharp decrease in enzyme stability, revealing the importance of subunits interaction for the stability of the enzyme. Another sensitive spot was found around position 345. Q345 forms 6 polar contacts with different residues from loop 177–183 (Fig 6B). This network, probably disrupted in the Q345K mutant, seems essential for enzyme stability. Likewise, the double mutation H172K/H220D revealed an important motif for the stability of the protein. Apparently, the intended K-D salt bridge caused no effect since the double mutant showed similar stability to the wild type. On the other hand, single mutations H172K and H220D both showed higher thermal sensitivity than the wild type (Fig 3A). A likely explanation is that the pair H172/H220 represents a His-His motif, which has been shown that increases protein stability [23–25]. The loss of the His-His interaction would be compensated by the introduction of the salt bridge.

The double mutant Q90R/Y509E showed significantly higher thermal stability than the wild-type enzyme (Fig 3A). However, the single mutations either did not cause a significant change (Y509E) or even decreased the stability of the enzyme (Q90R). Proximity of the two Gln 90 residues from both protein subunits at the dimer interface, may explain the instability of the Q90R mutant, due to electrostatic repulsion (S2 Fig). The double mutation allows the formation of a salt bridge between R90 and E509 (Fig 6C). The positive effect associated to this double mutation had an additive effect when it was combined with T554M. The triple mutant T554M/Q90R/Y509E showed a residual activity after heat treatment 2 to 3-fold higher than the wild-type enzyme. This increment is higher than any so far described for GOX [10,12,26].

## Supporting Information

S1 Fig Plasmid pSSP-GOX. (DOCX)

S2 Fig Location of the mutated residues in the GOX structure. (DOCX)

S1 File Procedure for GOX cloning and expression in yeast. (DOCX)
