# Supplementary material for: Identification and Structural Analysis of Amino Acid Substitutions that Increase the Stability and Activity of Aspergillus niger Glucose Oxidase
Source: PLoS One. 2015 Dec 7;10(12):e0144289. doi: 10.1371/journal.pone.0144289 (PMC4671603; doi:10.1371/journal.pone.0144289)

**S1 Fig**

Plasmid pSSP-GOX. The GOX coding sequence of A. niger excluding its signal peptide (AnGOX) is fused to the signal peptide from the glucoamylase STA1 from S. cerevisiae var. diastaticus (STA1sp), through a linker sequence containing the NheI restriction site. The whole fusion is cloned within the SacI/HindIII sites of pEMBLEYex4 and therefore it is under the control of the GAL10/CYC1 promoter (pGAL/CYC). The resulting plasmid encodes the URA3 gene and a defective LEU2 gene (dLEU2), complementing Ura and Leu auxotrophic requirements, respectively.


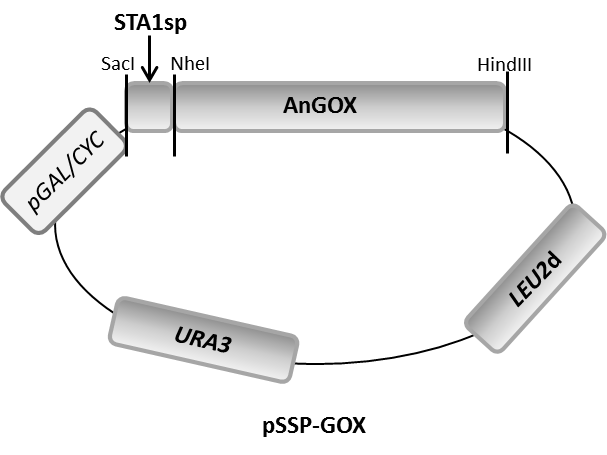

Supplement: S1 Fig — (DOCX) [file pone.0144289.s001.docx]
