# Supplementary material for: Identification and Structural Analysis of Amino Acid Substitutions that Increase the Stability and Activity of Aspergillus niger Glucose Oxidase
Source: PLoS One. 2015 Dec 7;10(12):e0144289. doi: 10.1371/journal.pone.0144289 (PMC4671603; doi:10.1371/journal.pone.0144289)

**S2 Fig.**

Location of the residues mutated in this study within the overall structure of GOX. Each of the monomers composing the dimer is shown in a different color (green or blue). The subunit of origin is indicated in brackets. The cofactor FAD is shown in red.


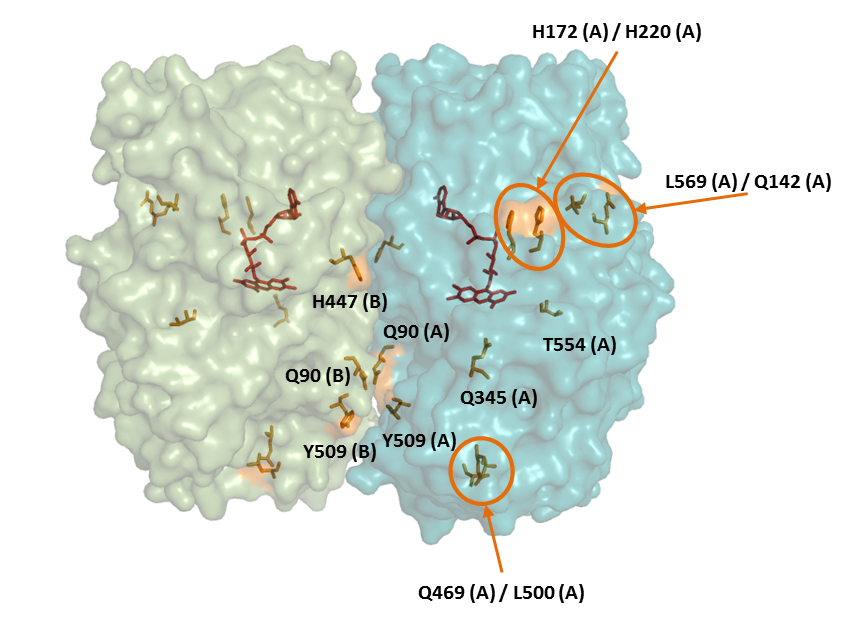

Supplement: S2 Fig — (DOCX) [file pone.0144289.s002.docx]
