# Supplementary material for: Identification and Structural Analysis of Amino Acid Substitutions that Increase the Stability and Activity of Aspergillus niger Glucose Oxidase
Source: PLoS One. 2015 Dec 7;10(12):e0144289. doi: 10.1371/journal.pone.0144289 (PMC4671603; doi:10.1371/journal.pone.0144289)
Supplement: S1 File — (DOCX) [file pone.0144289.s003.docx]

**GOX cloning and expression in yeast.**

Genomic DNA was isolated from A. niger CECT 2775 as follows: The biomass from a 5 mL culture grown for 48 hours was resuspended in 300 μl of TNES buffer (200 mM Tris pH 8.5, 250 mM NaCl, 25 mM EDTA, 0.5% SDS). Cells were lysed in a bead-beater with 5 steel beads of 2.3 mm diameter for 2 minutes at 4 ºC. The supernatant recovered after centrifugation at 12000 rpm for 10 minutes at 4ºC was mixed with 300 μl 3M NaOAc pH 5.2, and the precipitate was removed again by centrifugation in the same conditions. DNA was precipitated by addition of 1 volume of isopropanol. The pellet was washed with 200 μl of 70% ethanol, dried and finally resuspended in 50 μl of TE (10 mM Tris pH 8, 1 mM EDTA).

GOX coding sequence, excluding its signal peptide, was amplified by PCR with oligonucleotides JM823 (TACGCTAGCAGCAATGGCATTGAAGCCAGC) and JM824 (CACAAGCTTTCACTGCATGGAAGCATAATCTTC), containing the NheI and HindIII restriction sites, respectively (underlined). PCR was carried out with a proofreading polymerase (Phusion, Thermo Sci), according to the specifications of the manufacturer. All cloning experiments in E. coli were carried out using strain XL1-Blue (Agilent technologies).

Plasmid pSTRD-Bgl1 (Marin-Navarro et al 2010) was used to construct the expression vector for A. niger GOX. pSTRD-Bgl1 was derived from the yeast expression vector pEMBLYex4 and contained the N-terminal domain of STA1 from S. cerevisiae, including its signal peptide, fused to the coding

sequence of Bgl1. The N-terminal domain from STA1 was deleted from pSTRD-Bgl1 with oligonucleotides JM696 (ACCCAAAGCTGAGTTAAATAGAAGC) and SA639 (GCTGGTGCTAGCGGTGTCCCAATTCAAAACTATACCCAG), to generate the plasmid pSSP-Bgl1 where the STA1 signal peptide is fused to the Bgl1 coding sequence through a linker containing the NheI restriction site (underlined). This coding sequence was removed by digestion with NheI and HindIII and substituted by the GOX PCR product previously digested with the same enzymes. In the resulting plasmid designated pSSP-GOX (Figure S1) the GOX coding sequence is under the control of a promoter inducible by galactose and secretion is triggered by the STA1 signal peptide which was previously shown to be effective for heterologous protein expression in S. cerevisiae (Marin-Navarro et al 2010). The whole insert encoding the fusion protein was sequenced from two independent clones.
